# Supplementary material for: A Role for Human DNA Polymerase λ in Alternative Lengthening of Telomeres
Source: Int J Mol Sci. 2021 Feb 27;22(5):2365. doi: 10.3390/ijms22052365 (PMC7956399; doi:10.3390/ijms22052365)
Supplement: Supplementary file 1 [file ijms-22-02365-s001.pdf]

Supplementary Information to the manuscript:

A role for human DNA polymerase λ in alternative lengthening of telomeres

Elisa Mentegari [1#], Federica Bertoletti [1#], Miroslava Kissova [1#†], Elisa Zucca [1], Silvia Galli [1], Giulia Tagliavini [1], Anna Garbelli [1], Antonio Maffia [1], Silvia Bione [1], Elena Ferrari [2], Fabrizio d'Adda di Fagagna [1,3], Sofia Francia [1], Simone Sabbioneda [1], Liuh-Yow Chen [4], Joachim Lingner [4], Valerie Bergoglio [5], Jean-Sebastien Hoffmann [6], Ulrich Hübscher [2], Emmanuele Crespan [1*‡] and Giovanni Maga [1*‡]

[1] Institute of Molecular Genetics IGM-CNR "Luigi Luca Cavalli-Sforza", via Abbiategrasso 207, I-27100 Pavia (Italy); federica.bertoletti@igm.cnr.it (FB); miroslava.kissova@ntnu.no (MK), mentegari@igm.cnr.it (EM); elisa.zucca83@gmail.com (EZ); silvia.galli@cruk.cam.ac.uk (SG); g.tagliavini@sms.ed.ad.uk (GT); anna.garbelli@igm.cnr.it (AG); antonio.maffia@berkeley.edu (AM); bione@igm.cnr.it (SB); elena.ferrari@dmmd.uzh.ch (EF); fabrizio.dadda@ifom.eu (FAF); emmanuele.crespan@igm.cnr.it (EC); giovanni.maga@igm.cnr.it (GM)

[2] Department of Molecular Mechanisms of Disease, University of Zürich-Irchel, Winterthurerstrasse 190, CH-8057 Zürich (Switzerland); elena.ferrari@dmmd.uzh.ch (EF); hubscher@ uzh.ch (UH)

[3] IFOM-The FIRC Institute of Molecular Oncology, Milan 20139 (Italy)

[4] Swiss Institute for Experimental Cancer Research (ISREC), School of Life Sciences, Frontiers in Genetics National Center of Competence in Research, Ecole Polytechnique Fédérale de Lausanne (EPFL), Station 19, CH-1015 Lausanne (Switzerland). lyowchen@gate.sinica.edu.tw (L-YC); joachim.lingner@epfl.ch (JL)

[5] UMR1037 INSERM, Cancer Research Center of Toulouse, 2 Avenue Curien, 31037 Toulouse (France); valerie.bergoglio@inserm.fr

[6] Laboratoire d'Excellence Toulouse Cancer (TOUCAN), Laboratoire de Pathologie, Institut Universitaire du Cancer-Toulouse, Oncopole, 1 avenue Irène-Joliot-Curie, 31059 Toulouse Cedex, France ; jean-sebastien.hoffmann@inserm.fr

* Correspondence: giovanni.maga@igm.cnr.it (GM); emmanuele.crespan@igm.cnr.it (EC)

# These authors contributed equally to this work.

† Present address: Institute of Clinical and Molecular Medicine, Norwegian University of Science and Technology, Erling Skjalgssons gt 1, 7491 Trondheim, Norway

‡ These authors should be considered joint senior authors.

Contents:

Supplementary Figure Legends for Figures S1-S8

Supplementary Figures S1-S8

Supplementary Figure Legends

Supplementary Figure S1. A. MMST DNA synthesis by Pol λ in the presence of different combinations of dNTPs and in the presence of both the donor and acceptors strands (lanes 3 - 9) or with the donor strand alone (lanes 10-14). Lane 1, labelled 25 mer donor alone; lane 2, control reaction in the absence of dNTPs. **B.** Pol λ was incubated in the presence of dNTPs, (either single or in combination) with the 5'-labelled dideoxy-terminated16/48 acceptor template. Lane 1, 16 mer labelled oligonucleotide alone; lane 2, control reaction in the absence of dNTPs. **C**. MMST DNA synthesis by Pol β in the absence (lane 1) or in the presence (lanes 2-4) of different concentrations of RP-A and in the presence of the 5'-labelled donor strand and the dideoxy terminated 16/48mer acceptor template. Lane 5, control reaction with Pol λ. **D.** Titration of the five telomeric repeats 5xtel (lanes 1-5) or the single telomeric repeat (lanes 6-10) dideoxy terminated 16/48mer acceptor templates, in the presence of 50 nM Pol λ, RP-A and dGTP.

Supplementary Figure S2. A. Confocal microscopy imaging of Pol λ colocalization with TERRA RNA in Saos-2 cells ectopically expressing c-Myc Pol λ and the corresponding statistical analysis. The white arrow shows a representative colocalization event. **B**. Confocal microscopy imaging of Pol λ and TERRA foci in Saos-2 cells either transfected with the control empty vector (top and mid lane) or with the vector ectopically expressing c-Myc Pol λ

(bottom lane), untreated (top lane) or treated with RNase A before staining (mid and bottom lanes).

Supplementary Figure S3. Original uncropped images of the western blots shown in Fig.1A, lanes 1-4.

Supplementary Figure S4. Original uncropped images of the western blots shown in Fig.1A, lanes 5-6.

Supplementary Figure S5. Original image of the western blots shown in Fig.2H, lanes 1-2. area shown in Figure 2H is boxed.

Supplementary Figure S6. Original uncropped image of the gel shown in Fig. 4G, lanes 1-6. The area between the solid lines was cropped out in Fig. 4G. The omitted lanes represent another Pot1/TTP1 titration with a lower Pol λ concentration.

Supplementary Figure S7. Original uncropped image of the gel shown in Fig.4 H, lanes 1-8.

Supplementary Figure S8. Original uncropped image of the gel shown in Fig.4 E.

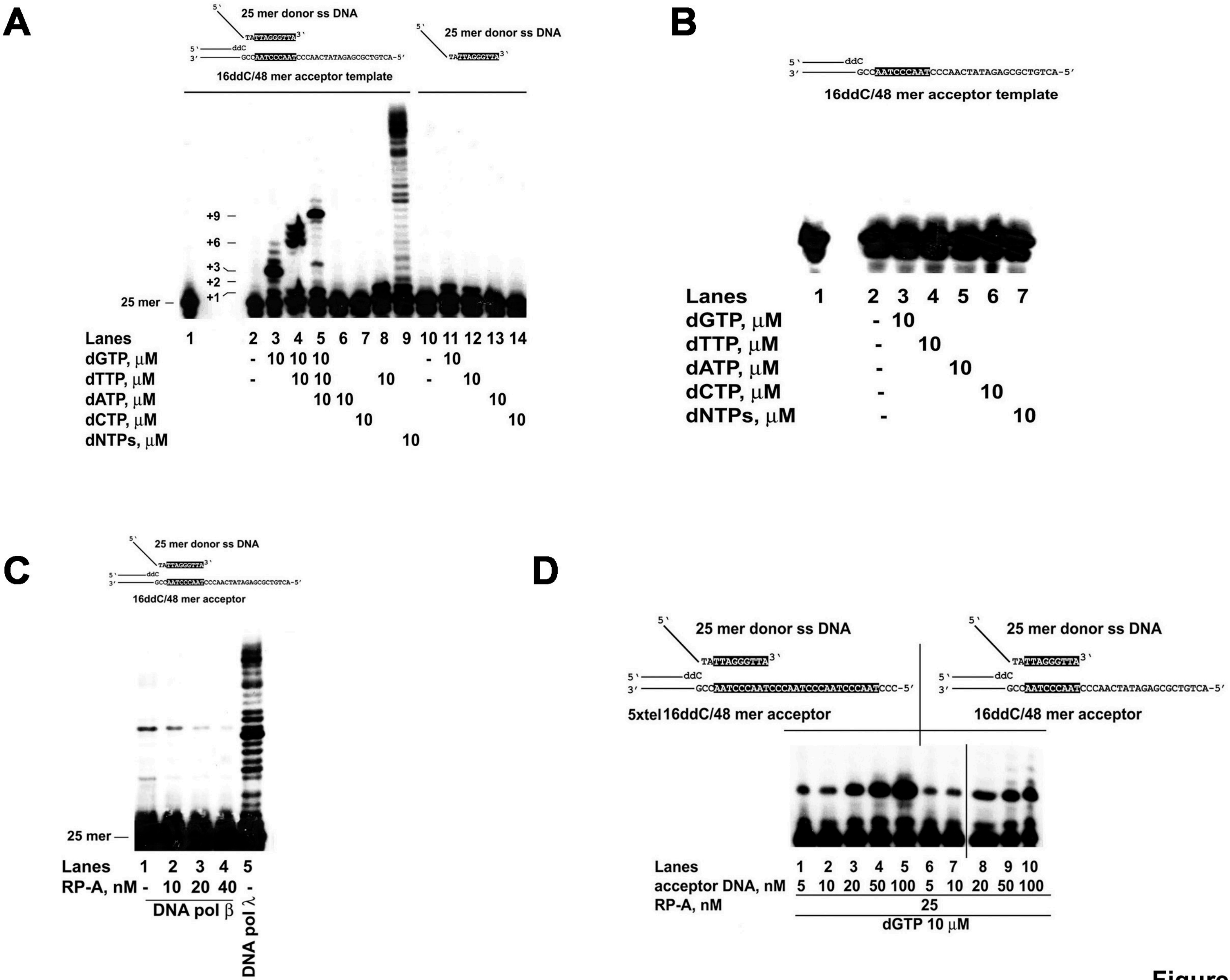
A
25 mer donor ss DNA
TATTAGGGTTA 3'
16ddC/48 mer acceptor template
+9
+6
+3
+2
+1
25 mer
Lanes 1 2 3 4 5 6 7 8 9 10 11 12 13 14
dGTP, μM
dTTP, μM
dATP, μM
dCTP, μM
dNTPs, μM
B
16ddC/48 mer acceptor template
Lanes 1 2 3 4 5 6 7
dGTP, μM - 10
dTTP, μM - 10
dATP, μM - 10
dCTP, μM - 10
dNTPs, μM - 10
C
25 mer donor ss DNA
16ddC/48 mer acceptor
25 mer
Lanes 1 2 3 4 5
RP-A, nM - 10 20 40 -
DNA pol β
DNA pol λ
D
25 mer donor ss DNA
5xtel16ddC/48 mer acceptor
16ddC/48 mer acceptor
Lanes 1 2 3 4 5 6 7 8 9 10
acceptor DNA, nM 5 10 20 50 100 5 10 20 50 100
RP-A, nM 25
dGTP 10 μM

Figure S1

A

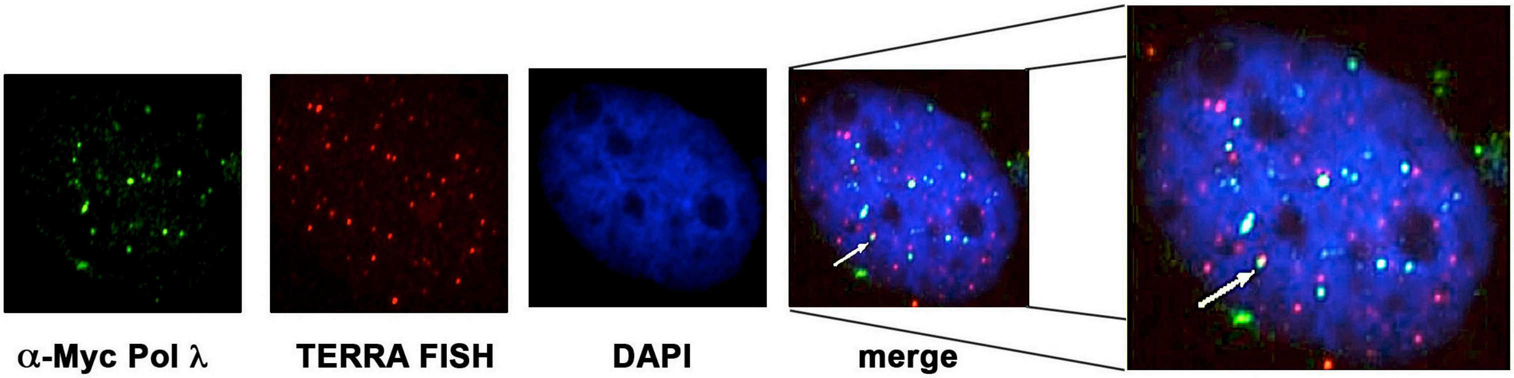

Cells	Pol λ foci (tot)	TPP1 foci (tot)	Colocalizations expected	Colocalizations observed	χ^2 *p*-value
21	404	1056	4.2	7	*p*=0.134

B

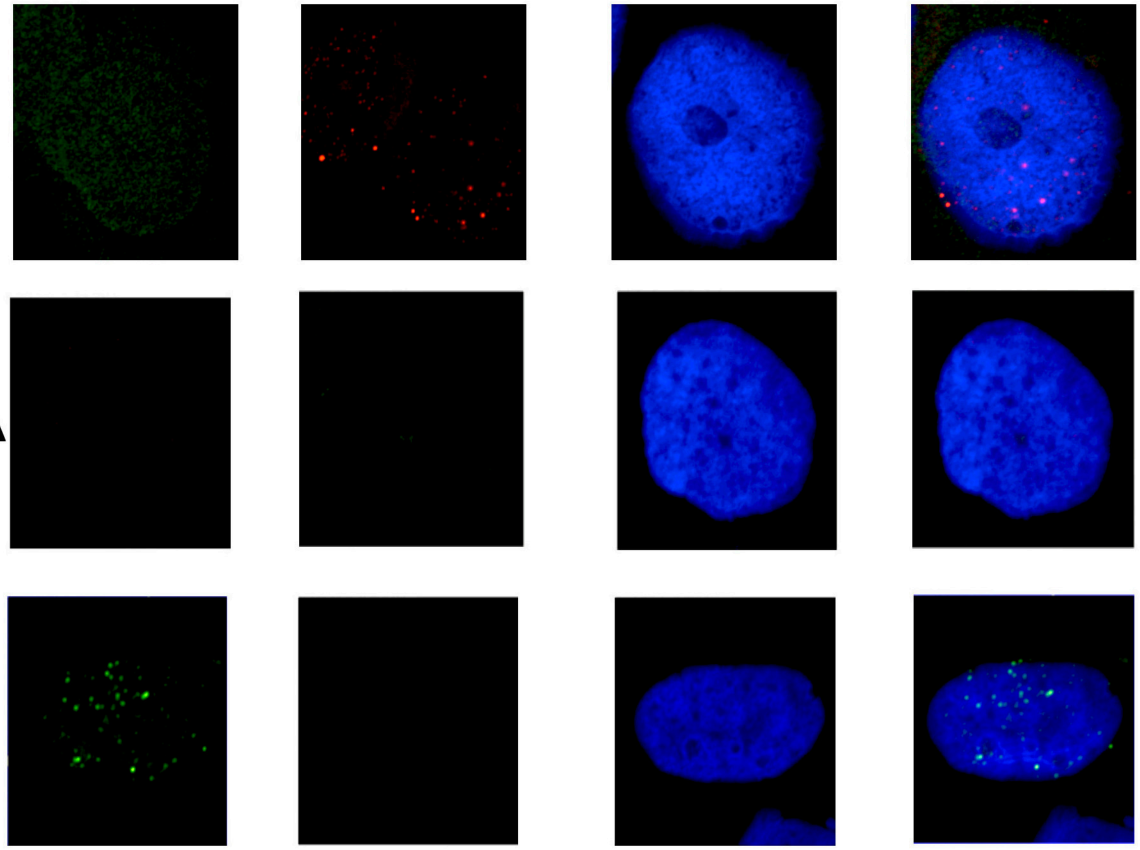

Figure S2

Fig 1 A, lanes 1-4

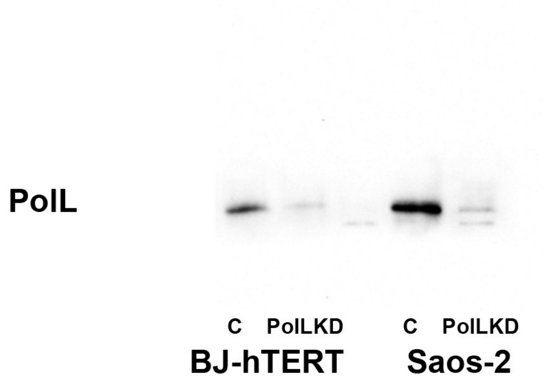

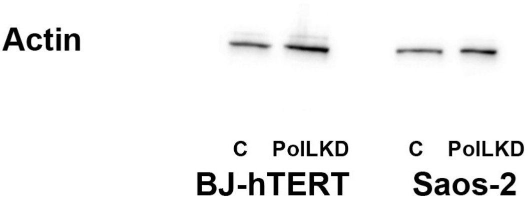

Figure S3

Fig.1A, lanes 5-6

Pol L

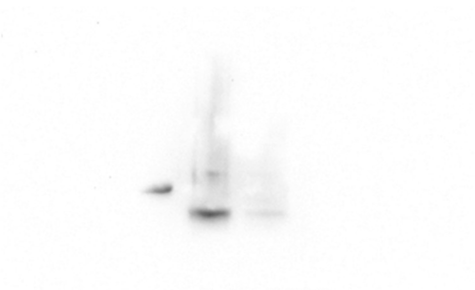

C PolLKD

U2OS

Actin

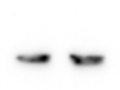

c PolLKD

U2OS

Figure S4

Fig.2H, lanes 1,2

22-01-19 chemidoc

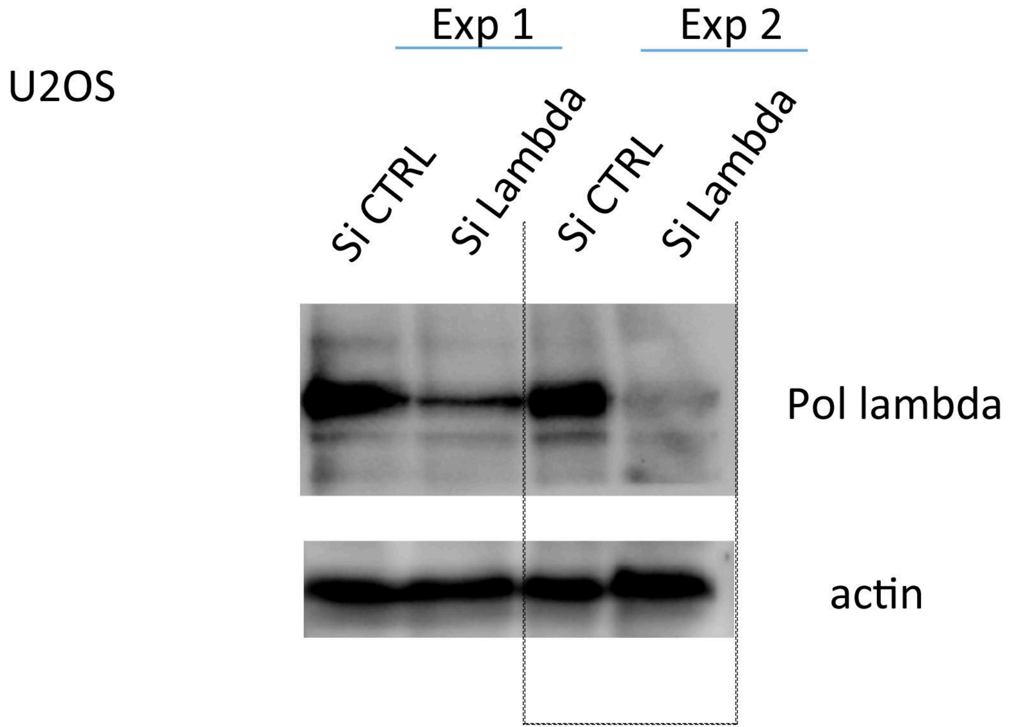

Figure S5

Fig. 4 G lanes 1, 2-6

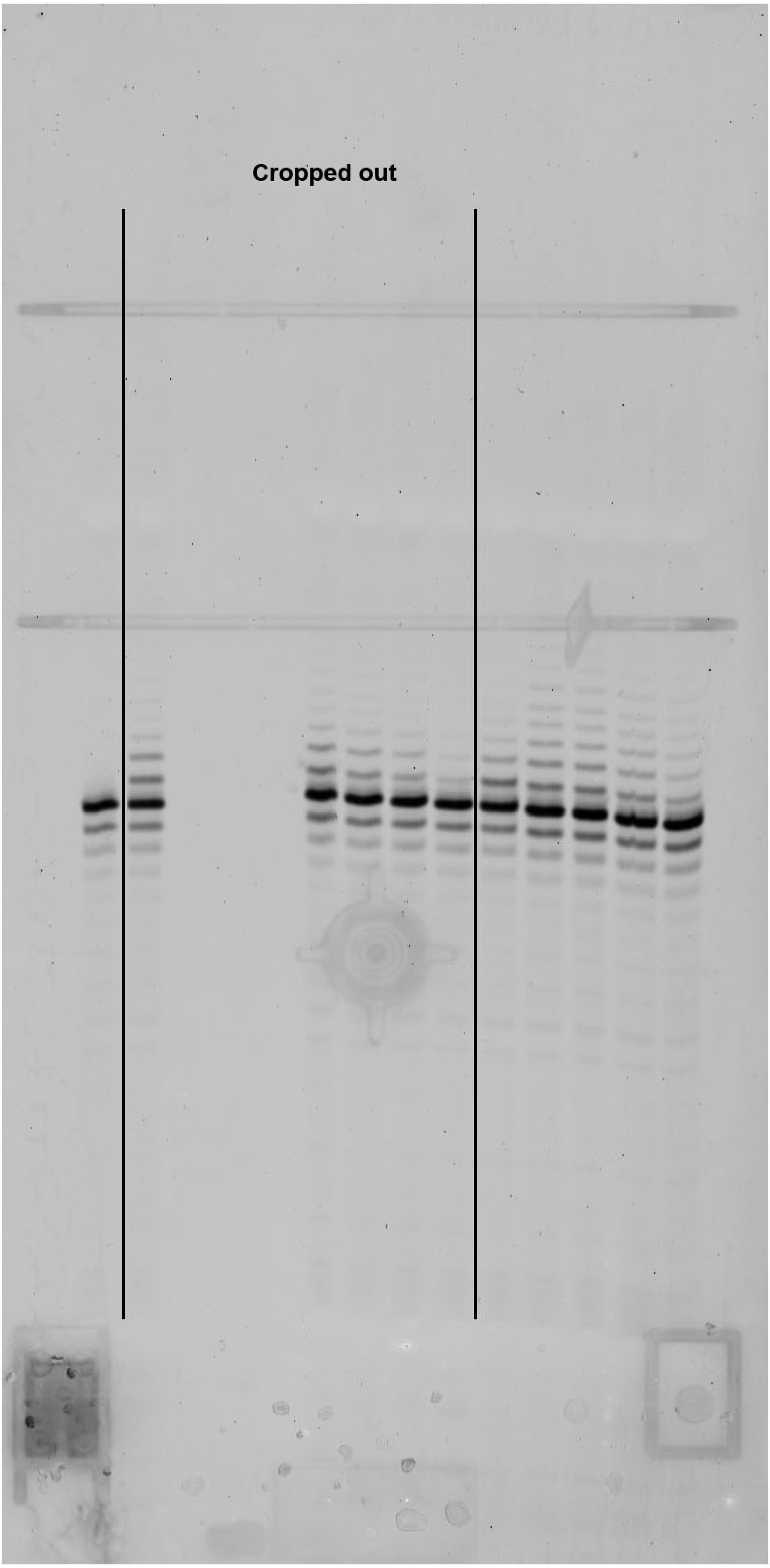

Figure S6

Fig. 4H, lanes 1-8

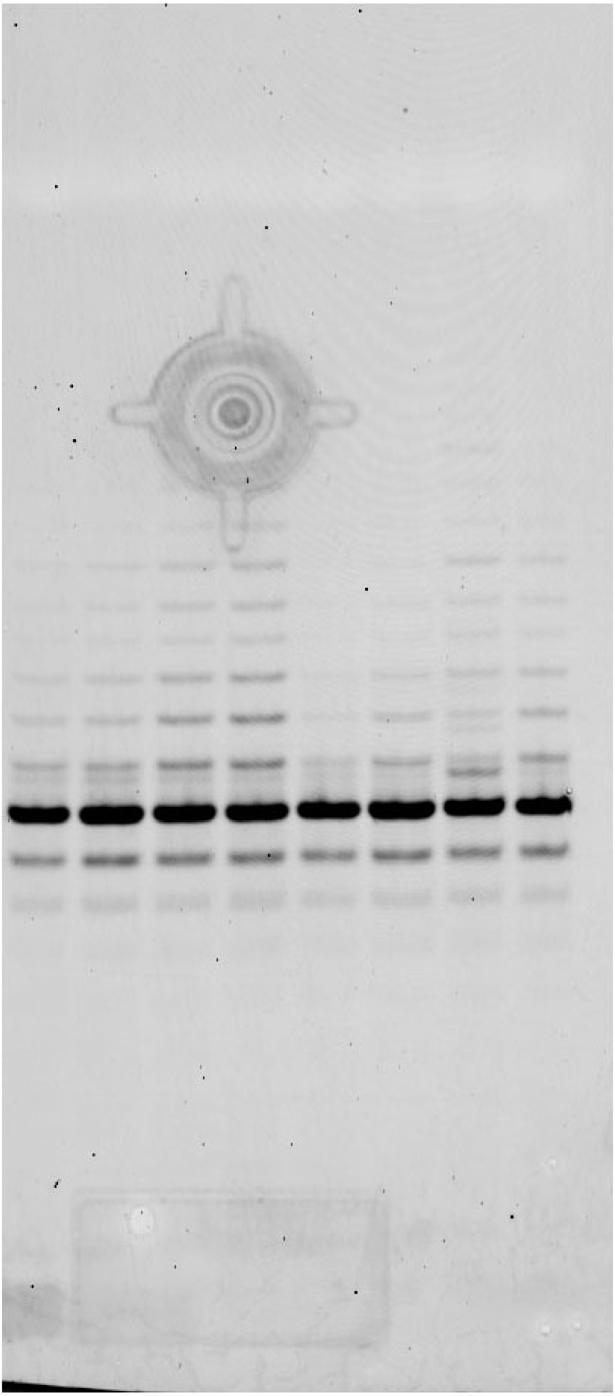

Figure S7

Fig 4 E

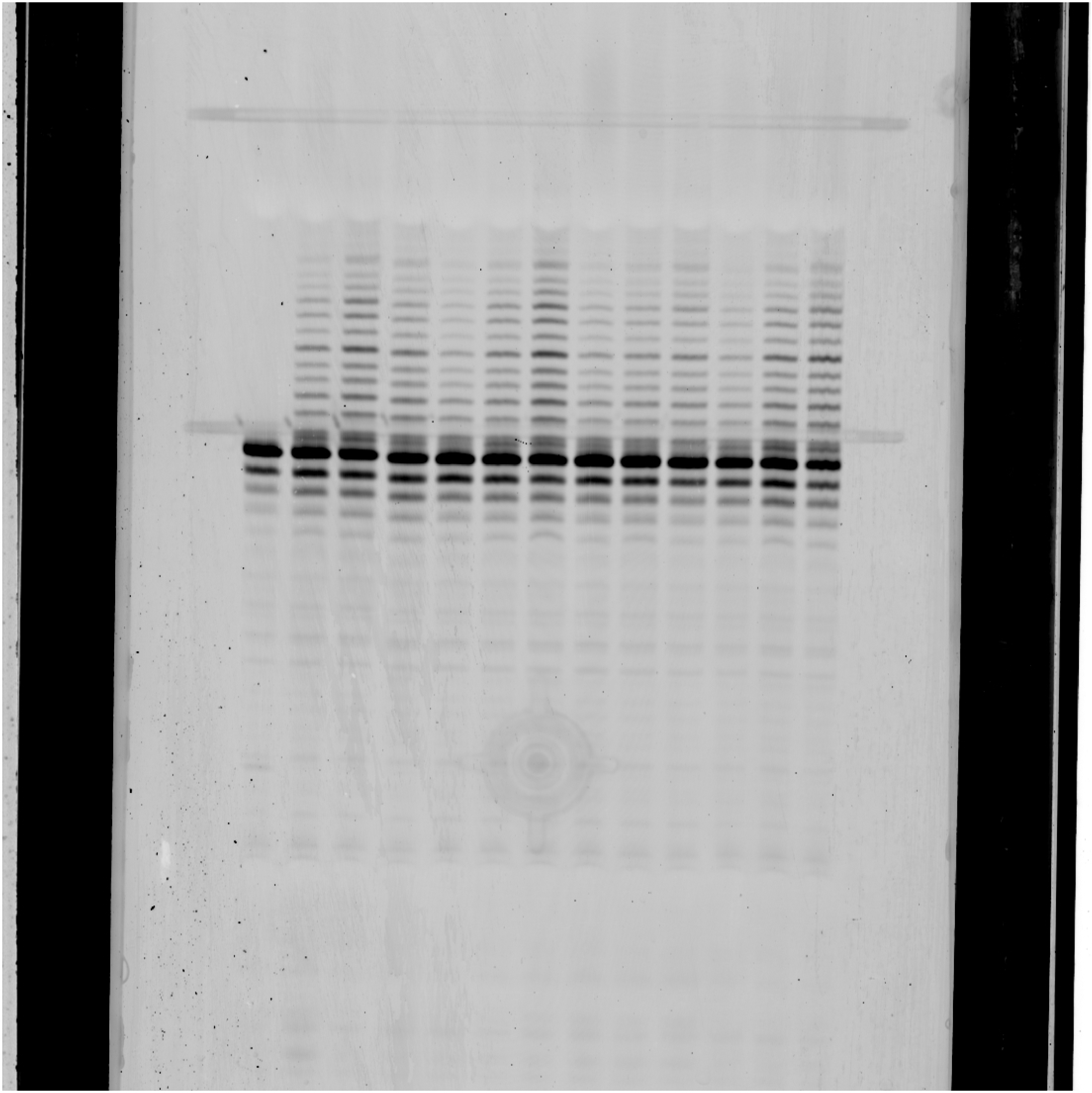

Figure S8
